# The Lunatics Gladdened

**Published:** 1860-12

**Authors:** 


### THE LUNATICS GLADDENED.

THERE are few of our readers, perhaps, who have not been laying away papers, and magazines, and books, for a more leisurely perusal, but that leisure time never comes, for every day brings up new and more pressing matter for reading and reflection quite as much as there is time for, even with diligence. With these views, we made a clean sweep of our library a fortnight ago, filling an immense box with magazines, monthlies, periodicals, reviews, pictorials, religious newspapers and books, which once upon a time we considered "precious," and sent them to the State Lunatic Asylum, at Auburn, New-York. We intended to have paid the freight, but the express-office sent them off contrary to orders, and then "couldn't tell any thing about it." The Superintendent received them duly, and thus writes: "Could you have seen for yourself how much comfort the patients have already derived from the papers alone, you would be amply repaid for your trouble." We sent the religious newspapers for the value of their contents and the variety of information, for variety suits the weak-minded and the feeble. We sent them agricultural exchanges, which we no more think of destroying than the religious papers, because the perusal of them would send their minds from present prisons and bars, and cells, to the sunnier times of childhood, and would "for the nonce," at least, make them happy in the remembrances which the sight of trees and cottages, and domestic animals, with the agricultural headings, would bring to them. We sent them, too, all the pictures we could find, for they interest all. Dear reader, go and do likewise, and pay the freight on what you send; let it be a "clean" present, and thus do something to happify the unfortunate ones whose intellects are clouded, and who are shut out from the great glad world, perhaps for life, for they once were as happy, and as glad, and as well as you are now, and as little as you thought that their last days would be spent in a mad-house.

Address, "Edward Hall, Auburn, New-York."

